# Small Paroxysmal Nocturnal Hemoglobinuria Clones in Autoimmune Hemolytic Anemia: Clinical Implications and Different Cytokine Patterns in Positive and Negative Patients

**DOI:** 10.3389/fimmu.2020.01006

**Published:** 2020-06-04

**Authors:** Bruno Fattizzo, Juri Giannotta, Anna Zaninoni, Austin Kulasekararaj, Lilla Cro, Wilma Barcellini

**Affiliations:** ^1^UO Ematologia, Fondazione IRCCS Ca' Granda Ospedale Maggiore Policlinico, Milan, Italy; ^2^Hematology Unit, King's College Hospital, London, United Kingdom; ^3^Servizio di Citofluorometria, Laboratorio Centrale, Fondazione IRCCS Ca' Granda Ospedale Maggiore Policlinico, Milan, Italy; ^4^Facoltà di Medicina e Chirurgia, Università degli Studi di Milan, Milan, Italy

**Keywords:** warm autoimmune hemolytic anemia, cold agglutinin disease, paroxysmal nocturnal hemoglobinuria, rituximab, cytokines

## Abstract

Autoimmune hemolytic anemia (AIHA) is characterized by immune mediated erythrocytes destruction by autoantibodies with or without complement activation. Additional pathologic mechanisms include cellular cytotoxicity, cytokline dysregulation, and inadequate bone marrow compensation with fibrosis/dyserythropoiesis. The latter resembles that of bone marrow failures, namely aplastic anemia and myelodysplastic syndromes. Paroxysmal nocturnal hemoglobinuria (PNH) clones are increasingly recognized in bone marrow failure syndromes, and their selection and expansion are thought to be mediated by immune mechanisms. In this study, we aimed to evaluate the prevalence of PNH clones in 99 patients with primary AIHA, and their correlations with disease features and outcomes. Moreover, in the attempt to disclose the physiopathology of PNH positivity in AIHA, serum levels of several immunomodulatory cytokines were tested. A PNH clone was found in 37 AIHA patients (37,4%), with a median size of 0.2% on granulocytes (range 0.03–85). Two patients showed a large clone (16 and 85%) and were therefore considered as AIHA/PNH association and not included in further analysis. Compared to PNH negative, PNH positive cases displayed a higher hemolytic pattern with adequate bone marrow compensation. AIHA type, response to therapy, complications and outcome were comparable between the two groups. Regarding cytokine levels, IFN-γ and IL-17 were lower in PNH positive vs. PNH negative AIHAs (0.3 ± 0.2 vs. 1.33 ± 2.5; 0.15 ± 0.3 vs. 3,7 ± 9.1, respectively, *p* = 0.07 for both). In PNH positive AIHAs, IFN-γ positively correlated with reticulocytes (*r* = 0.52, *p* = 0.01) and with the bone marrow responsiveness index (*r* = 0.69, *p* = 0.002). Conversely, IL-6 and IL-10 showed the same pattern in PNH positive and PNH negative AIHAs. IL-6 levels and TGF-β positively correlated with clone size (*r* = 0.35, *p* = 0.007, and *r* = 0.38, *p* = 0.05, respectively), as well as with LDH values (*r* = 0.69, *p* = 0.0003, and *r* = 0.34, *p* = 0.07, respectively). These data suggest testing PNH clones in AIHA since their prevalence is not negligible, and may correlate with a prominent hemolytic pattern, a higher thrombotic risk, and a different therapy indication. PNH testing is particularly advisable in complex cases with inadequate response to AIHA-specific therapy. Cytokine patterns of PNH positive and negative AIHAs may give hints about the pathogenesis of highly hemolytic AIHA.

## Introduction

Autoimmune hemolytic anemia (AIHA) is a clinically heterogeneous disease ranging from mild/ compensated to very severe life-threatening hemolysis (1, 2). Several pathogenic mechanisms are involved, mainly encompassing autoantibodies of different classes, thermal amplitude, and affinity/efficiency in activating complement. Other factors include abnormalities of antigen-presenting cells, increased antibody-dependent cellular cytotoxicity (ADCC) and cytotoxic CD8+T cells, aberrant cytokine production and inflammation, and alterations of immunoregulatory T cells. While direct complement-mediated lysis takes place mainly in the circulations and liver, ADCC and phagocytosis occur preferentially in the spleen and lymphoid organs (3). Finally, the efficacy of the erythroblastic compensatory response can greatly influence the clinical picture of AIHA (4–6). Intravascular hemolysis has been shown to correlate with the rate of complement activation and with the risk of AIHA related thrombosis. Bone marrow compensation has been demonstated to contribute to anemia severity at onset, the major predictor of disease relapse and outcome. Paroxysmal nocturnal hemoglobinuria (PNH) is a rare (incidence of 2–6 per million) clonal acquired disease, whose clinical spectrum includes both overt intravascular hemolysis and bone marrow failure, namely aplastic anemia (AA) and hypocellular myelodysplastic syndromes (MDS). PNH is due to a somatic mutation of the hematopoietic stem cell involving the PIG-A gene and resulting in deficiency of the complement inhibitory GPI-anchored proteins CD55 and CD59 (7–9). PNH has been classified by the International PNH Interest Group (IPIG) (8, 10) in three clinical subgroups: classic, PNH in the setting of another bone marrow disorder, and subclinical. The classic form is dominated by intravascular hemolysis, with markedly elevated LDH and a clone size >50%. The second group, mainly AA or MDS associated, is clinically dominated by the underlying bone marrow features and displays a clone size 10–50%. The third category is defined as subclinical, since there is no clinical or biochemical evidence of intravascular hemolysis and the PNH clone is <10%. Since the last 10 years, the increased sensitivity of the cytofluorimetric techniques (up to ≥0.01% clone size) enabled the detection of small PNH clones in up to 60% of AA and 30% of MDS (7–9, 11). The significance of these clones is still unclear, with some evidences for better response to immunosuppressive therapy in PNH positive cases. These tests allowed the detection of small PNH populations even in diseases not commonly associated to PNH such as hypomegakaryocytic thrombocytopenia (12) and chronic benign neutropenia (13). These forms are characterized by both immune mediated peripheral cytopenia and bone marrow failure, similarly to what demonstrated for AA and AIHA itself. We therefore analyzed the presence of small PNH clones in primary AIHA in order to evaluate their clinical significance in this acquired hemolytic disease. Furthermore, given that PNH typically arises in the context of autoimmune activation, we investigated immunoregolatory and inflammatory cytokines to address the role of PNH clones in the pathogenesis of AIHA.

## Methods

Ninety-nine patients with primary AIHA tested at Fondazione IRCCS Ca' Granda Ospedale Policlinico of Milan from March 2001 until October 2019 were included in the analysis. Demographic and clinical phenotypes were retrospectively evaluated from August 2017 until January 2020.

Clinical history, blood counts, hemolytic markers, AIHA type, bone marrow features, number and type of therapeutic interventions and their response rates, occurrence of complications (particularly thrombosis), and death were collected.

Primary AIHA was defined by hemolytic anemia and positive direct antiglobulin test (DAT), in the absence of associated overt lymphoproliferative, infectious, autoimmune, or neoplastic diseases. Patients were classified as wAIHA (DAT positive for IgG or IgG+C), CAD (DAT positive for C only, with high titer cold agglutinins), mixed (DAT positive for IgG+C with high titer cold agglutinins) and atypical (DAT negative, DAT positive for IgA only, warm IgM).

Reticulocytosis was evaluated and expressed as bone marrow responsiveness index (BMRI: absolute reticulocyte count x patient' Hb/normal Hb) (1).

### PNH Clone Testing

PNH testing had been performed by classical cytometry technique until 2010 and, thereafter, using high sensitivity (≥0.01%) fluorescent aerolysin (FLAER)-based assay according to 2010 International Clinical Cytometry Society (ICCS) PNH Consensus Guidelines and 2012 Practical PNH Guidelines (14, 15). FLAER/CD33/CD15/CD45 and FLAER/CD59 panels had been used for white blood cell (WBC) and red blood cell (RBC) testing, respectively.

### Evaluation of Immunomodulatory and Inflammatory Cytokines

In a fraction of patients (*N* = 11) the following cytokines were evaluated in serum using commercial ELISA kits (High Sensitivity Elisa kits, Invitrogen by Thermo Fisher Scientific, MA, USA, human TGF-β elisa kit, Immunological Sciences, Rome, Italy): interleukin (IL)6, IL10, IL17, tumor necrosis factor (TNF)-α, interferon (IFN)-γ, and transforming growth factor (TGF)-β. Cytokine levels were compared with 40 age and sex matched healthy controls.

### Statistical Analysis

Student *t*–test was used for continuous variables and chi-square test for categorical ones. Analysis of variance was performed by using mean, median, ranges and standard errors. Cumulative incidence of relapse, as well as overall survival, was evaluated by Kaplan Meyer method.

## Results

### Demographics and Hematologic Parameters

Clinical and hematologic features are shown in Table 1: 46% of patients were older than 60 years of age, male to female ratio was 0.98, and all AIHA types were represented (wAIHA, cAIHA, wAIHA+C, mixed and atypical cases). One third of cases presented severe anemia and hemoglobin levels positively correlated with LDH > 1.5 x ULN (*r* = 0.21 *p* = 0.03), indicating active intravascular hemolysis, as well as with inadequate reticulocytosis (i.e., BMRI <121, *N* = 22, *r* = 0.19, *p* = 0.05). Bone marrow evaluation had been performed in 74 cases and showed hypercellularity and diserythropoiesis in about half of cases (52 and 57%, respectively), and reticulin fibrosis (MF-1) in 42%; the latter displayed reduced BMRI compared with MF-0 patients (107 vs. 137, *p* = 0.05). Moreover, 63% of patients had a lymphoid infiltrate, with mainly T or mixed phenotype, not diagnostic for overt lymphoproliferative syndromes.

**Table 1:** Clinical and hematologic characteristics of AIHA patients, altogether and according to PNH positivity.

	**All patients**	**PNH neg**	**PNH pos**
**Clinical characteristics**	***N* = 99**	***N* = 62**	***N* = 37**
Median Age y(range)	57 (5–89)	57 (20–85)	63 (5–89)
M/F	49/50	30/32	20/17
Median follow up m(range)	20 (0–262)	26 (0–205)	24 (2–262)
WAIHA N(%)	37 (38)	27 (43.5)	10 (28.6)
WAIHA IgG+C N(%)	15 (16)	9 (14.5)	6 (17)
CAD N(%)	33 (34)	19 (31)	14 (40)
Mixed N(%)	5 (5)	3 (5)	2 (6)
Atypical N(%)	7(7)	4 (6.5)	3 (8.6)
Median Hb g/dL (range)	7.9 (1.4–13.7)	7.8 (3.5–13.1)	7.9 (1.4–13.7)
Median LDH U/L(range)	451 (150–3,200)	392 (150–1,867)	606 (191–3,200)^*^
Median LDH ULN (range)	2 (0–14)	2 (0–7)	2 (1–14)^**^
Median Ret x10^3^/mmc (range)	156 (5–574)	151 (5–478)	195 (38–574)
Median BMRI (range)	103 (2–378)	95 (2–305)	128 (18–378)
BMRI <121 N(%)	52 (52)	38 (61)	14 (38)^£^
**Bone marrow evaluation**	***N*** **=** **74**	***N*** **=** **47**	***N*** **=** **26**
Median Cellularity % (range)	55 (15–100)	55 (20–100)	55 (15–95)
Hypercellularity (%)	38 (51)	24 (51)	14 (54)
Fibrosis MF1 (%)	31 (42)	22 (46)	9 (35)
Dyserythropoiesis (%)	42 (57)	28 (58)	14 (52)
Median lymphoid infiltrate%(range)	5 (0–75)	5 (0–75)	5 (0–30)
Type of infiltrate B(%)	10 (10)	8 (13)	2 (6)
T(%)	28 (29)	16 (26)	12 (34)
Mixed (%)	23 (24)	16 (26)	7 (20)

*PNH paroxysmal nocturnal hemoglobinuria; CAD cold agglutinin disease; WAIHA warm autoimmune haemolytic anemia; Hb hemoglobin; Ret reticulocytes; LDH lactate dehydrogenase; ULN upper limit of normality; BMRI bone marrow responsiveness index*.

* *P = 0.005*;

** *P = 0.03*;

£ *P = 0.01*.

### AIHA Treatment, Complication and Outcome

Thirty-nine (39%) of patients required at least one transfusion during the follow up, and 94% received AIHA treatment (Table 2). Specifically, 89% were treated with steroids, 71% responded, and 55% relapsed and required further treatment. Second line therapy included rituximab (57% with an overall response rate of 81.5%), splenectomy (7.2% with 75% responsive cases), and cytotoxic immunosuppressants (20.8%, with a response in 65% of patients). On the whole, patients received a median of 2 (range 0–5) therapy lines. Regarding AIHA related complications, 7% of cases developed acute renal failure, 31% an infectious episode, 12% experienced a thrombosis (2 pulmonary embolisms and 10 lower limbs deep venous thrombosis), and 8% died (3 patients for AIHA-related complications).

**Table 2:** Treatments and outcome of AIHA patients, altogether and according to PNH positivity.

	**All patients**	**PNH neg**	**PNH pos**
**Treatment and outcome**	***N* = 99**	***N* = 62**	***N* = 37**
First therapy line *N*(%)	96 (96)	61(98)	35 (94)
Second therapy line *N*(%)	57 (57)	35 (56)	20 (54)
Third therapy line or > *N*(%)	31 (31)	19 (31)	12 (32)
Median RFS days (range)	539 (25–6,014)	700 (25–6,014)	338 (42–3,483)
Evans (%)	14 (14)	6 (10)	7 (19)
Acute renal failure (%)	7 (7)	6 (10)	1 (3)
Infections (%)	31 (31)	19 (31)	10 (29)
Thrombosis (%)	12 (12)	7 (11)	5 (14)
Death (%)	8 (8)	3(5)	5(14)
Median OS m(%)	25 (0–262)	26 (0–205)	24 (2–62)

*OS, overall survival; RFS, relapse free survival*.

### PNH Clone Analysis and Description of Two Peculiar Cases

**Figure 1:**
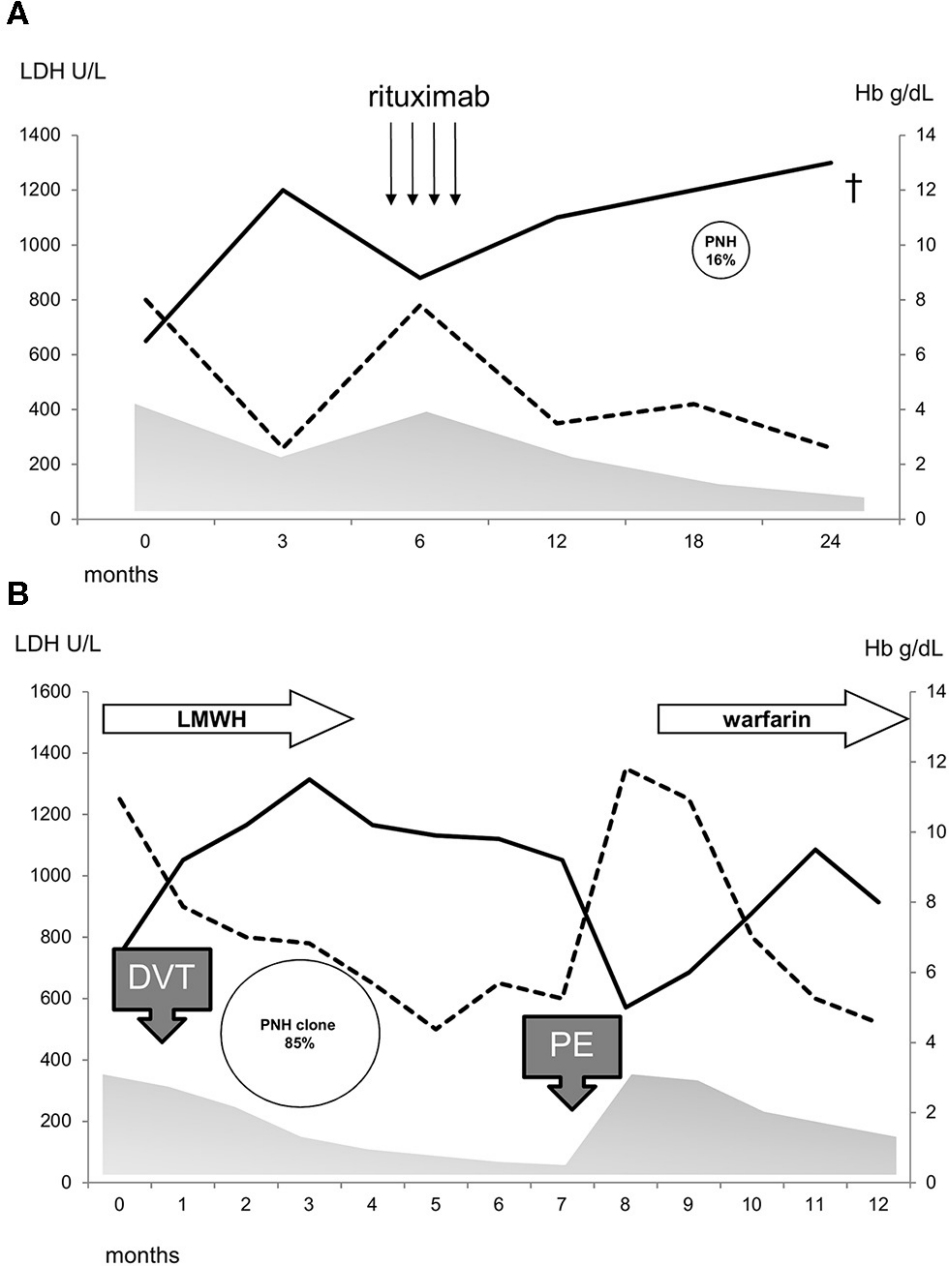
Clinical course of two patients **(A,B)** with PNH/AIHA association and a clone size>10%. Hb, continuous line; LDH, dotted line; gray area, prednisone therapy; arrows, rituximab 375 mg/sm/week for 4 weeks; LMWH, low molecular weight heparin; thrombosis, DVT, deep venous thrombosis; PE, pulmonary embolism; cross indicates death.

Thirty-seven cases (37%) showed a PNH clone on granulocytes. Five patients had been tested before FLAER era (2 showed a PNH clone size of 0.2% on granulocytes) and 3 of them were re-evaluated thereafter (all PNH negative). PNH positive AIHA showed increased LDH levels as compared to negative ones (*p* = 0.005) and mostly adequate reticulocytosis (BMRI>121 in 62% vs. 39% in PNH negative, *p* = 0.01). Other hematologic features, including AIHA type, were comparable among the two groups (Table 1). Notably, relapse free survival (RFS) after steroids was slightly shorter in PNH positive than in negative cases, whilst no other differences in treatment choice or response rate were noted. In PNH positive patients, median clone size on granulocytes was 0.2% (0.03–85). Only two patients displayed a PNH clone >10% and both showed LDH levels >1.5xULN. The first patient was a 40-year-old man, initially diagnosed with primary wAIHA that was effectively treated with steroids and rituximab; subsequently a PNH clone 16% was detected and he developed a severe and fatal *Pneumocystis jerovecii* pneumonia (Figure 1A). The second patient was a 65-year-old lady diagnosed with very severe wAIHA responsive to steroids with amelioration of anemia. However, LDH levels were persistently high, and a lower limb venous thrombosis occurred. Re-evaluation of other causes of hemolysis, including congenital, toxic, mechanical, and infective forms, demonstrated a PNH clone 85% on granulocytes (Figure 1B). The patient started low molecular weight heparin, but after discharge discontinued treatment. She presented 2 months later with a massive pulmonary embolism and very severe haemolytic anemia (Hb 4.2 g/dL and LDH 5.7xULN). DAT tube was still positive and PNH clone unchanged. She restarted anticoagulation, was transfused, and commenced eculizumab. Since these two cases resemble PNH (subclinical and haemolytic type, respectively), were not included in further correlations.

### Cytokine Studies

**Figure 2:**
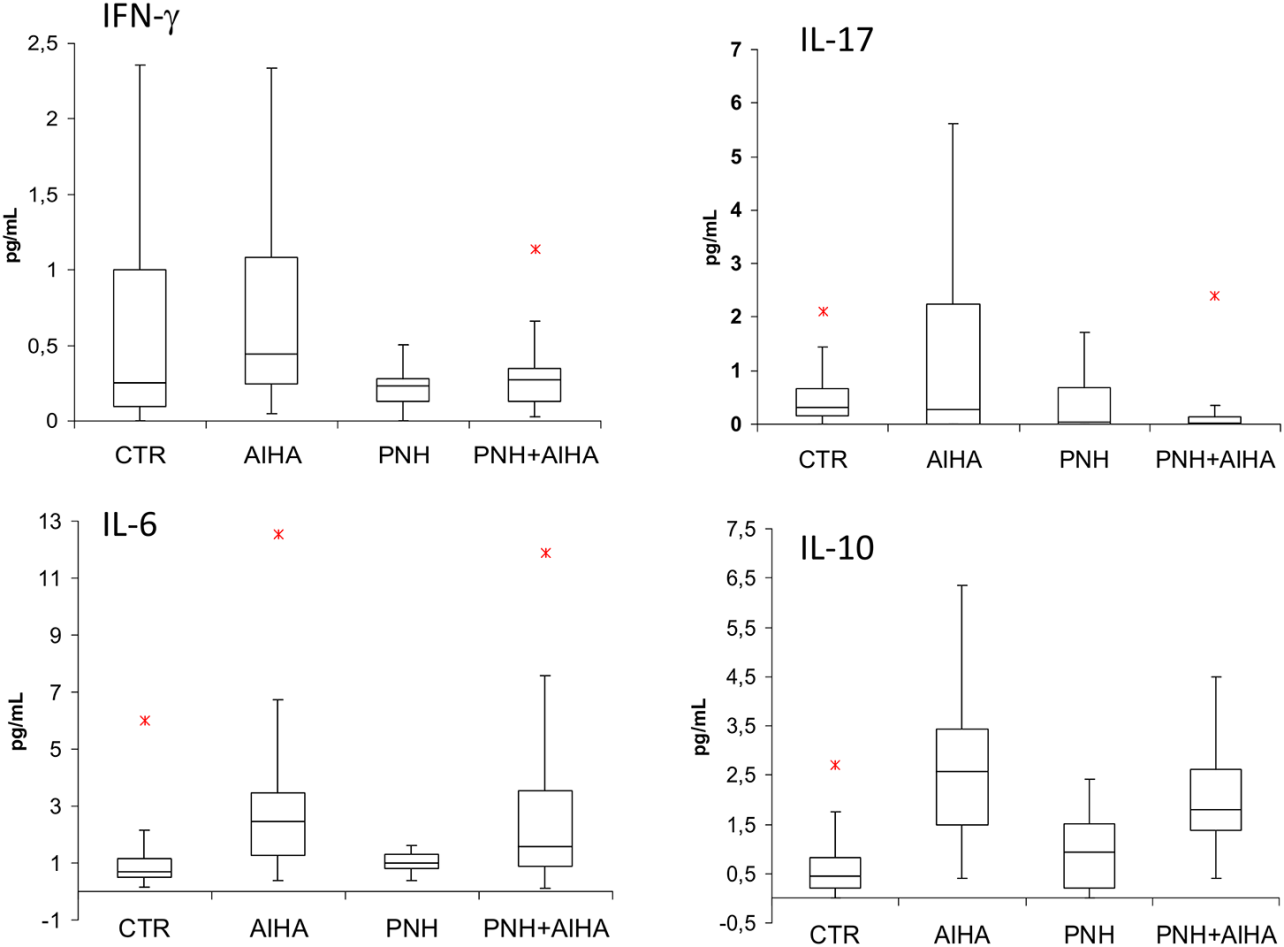
cytokine levels in PNH positive and PNH negative AIHA patients, in age and sex matched controls (*N* = 40), and in a cohort of classic hemolytic PNH cases (*N* = 28). IFN-γ and IL-17 levels were lower in PNH positive versus PNH negative AIHA (*p* = 0.07 for both). No significant p values were obtained. TNF-α and TGF-β levels are not shown into the figure; their values were (mean ± SD): TNF-α 0.2 ± 0.2 pg/mL in PNH+AIHA, 0.2 ± 0.1 pg/mL in PNH-AIHA, 0.23 ± 0.29 pg/mL in classic PNH, and 1.3 ± 0.9 pg/mL in healthy controls. TGF-β 3,249 ± 1,570 pg/mL in PNH+AIHA, 3,295 ± 1,697 pg/mL in PNH-AIHA, 21,010 ± 602 pg/mL in classic PNH, and 3,160 ± 1,884 pg/mL in healthy controls. *represent outlier values.

Figure 2 shows cytokine levels in PNH positive and PNH negative AIHA patients, in age and sex matched controls (*N* = 40), and in a cohort of classic hemolytic PNH cases (*N* = 28). IFN-γ and IL-17 levels were lower in PNH positive vs. PNH negative AIHA (0.3 ± 0.2 vs. 1.33 ± 2.5; 0.15 ± 0.3 vs. 3,7 ± 9.1, respectively, *p* = 0.07 for both), whilst IL-6 and IL-10 were not significantly different in the two groups.

As compared to healthy controls, IFN-γ and IL-17 levels were reduced in PNH positive AIHA, similarly to what observed in patients with classical PNH. Conversely, IL-6 and IL-10 were greater compared to healthy volunteers. Concerning TNF-α and TGF-β levels, no clear differences emerged among groups. Focusing on PNH positive AIHA patients, IFN-γ positively correlated with reticulocytes (*r* = 0.52, *p* = 0.01) whilst IL-17 showed a negative correlation (*r* = −0.4, *p* = 0.04). Similar results were observed for the bone marrow responsiveness index (*r* = 0.69 for IFN-γ, *p* = 0.002 and *r* = −0.40 for IL-17, *p* = 0.04). IL-6 levels and TGF-β positively correlated with clone size (*r* = 0.35, *p* = 0.007, and *r* = 0.38, *p* = 0.05, respectively), as well as with LDH values (*r* = 0.69, *p* = 0.0003, and *r* = 0.34, *p* = 0.07, respectively). Finally, TNFα levels showed a negative correlation with clone size (*r* = −0.4, *p* = 0.03).

## Discussion

In this study we showed for the first time the presence of a PNH clone in about one third of 99 consecutive AIHA patients. Almost all cases displayed a small clone (<10%) considered the hallmark of subclinical PNH according to IPIG classification (8, 10). Two patients showed a greater clone size and were therefore diagnosed as PNH associated with AIHA. This association has not been previously described and should be considered when the patient is referred for hemolytic anemia. In fact, thrombotic events are known to occur in about 10% of active AIHA, whilst the frequency is far higher (about 40%) in classic hemolytic PNH (8, 16). Of note, in one of the two AIHA/PNH patients, a severe thrombotic event occurred soon after the discontinuation of anticoagulant prophylaxis.

An interesting point is the clinical significance of small PNH clones in the remaining AIHA studied patients. It is known that small PNH clones are present in more than 50 and 20% of patients with aplastic anemia or hypoplastic MDS, respectively (8, 11, 16). Their clinical significance is still a matter of debate, although in various series an association with a deeper cytopenia, increased LDH levels, and thrombotic tendency have been demonstrated in PNH positive cases (11). Moreover, a better response to immunosuppressive treatment was reported in this group, stressing the link between PNH clone emergence and immune mediated bone marrow failure. Among the hypothesis of PNH clone selection in the context of bone marrow failure, it has been reported that GPI molecules may be the target of the autoimmune attack; therefore, GPI negative stem cells are spared and may further expand through still unknown mechanisms, including additional mutations and environmental factors (8, 9, 16). Our results showed that PNH positive AIHA cases had greater LDH levels, suggesting a higher hemolytic pattern and possibly an increased thrombotic risk, although the number of cases and the follow up may be insufficient to draw definitive conclusions. As regards therapy outcome, no clear associations have been demonstrated with PNH positivity in this AIHA cohort. Another feature of PNH positive AIHAs is their better bone marrow compensatory response compared with PNH negative cases. Consistently, hemoglobin values were comparable between the two groups, in spite of the more marked hemolytic pattern observed in the former.

On the whole, despite the few clinical correlations found possibly due to the small sample size and relative short follow-up, PNH clone testing may be included in the initial work up of AIHA to either assess the presence of the two diseases requiring different and specific therapy. Moreover, PNH clone testing is advisable in complex cases with inadequate response to AIHA-specific therapy and with persistent hemolytic activity.

In the attempt to investigate the physiopathology underlying the emergence of PNH clones in AIHA, we tested several immunomodulatory and inflammatory cytokines. PNH positive AIHA patients showed an immunological signature distinct from negative cases, with reduced levels of IFN-γ and IL-17. The former is a classic T-helper 1 cytokine involved in the autoimmune attack against bone marrow precursors typical of aplastic anemia (3) and reported elevated also in AIHA. Likewise, IL-17, a cytokine known to amplify the pro-inflammatory and autoimmune response, has been reported elevated in the same settings (17–19). In our study, PNH positive AIHA showed a Th1 profile more similar to hemolytic PNH than to “classic” (PNH negative) AIHA, suggesting that these small clones might mitigate Th1 response. On the contrary, Th2 cytokines levels, i.e., IL-6 and IL-10, were not different in PNH positive and negative AIHA cases. This finding suggests that Th2 signature, the hallmark of humoral autoimmunity, is particularly strong in AIHA and is not influenced by the presence of a small PNH clone. Although these data are preliminary and would need further *ad-hoc* studies, it is tempting to speculate that the detection of a small PNH clone in AIHA reveals the presence of a wider spectrum of immunologic mechanisms involved in pathogenesis of the disease compared with PNH-negative AIHAs. In fact, in the PNH-positive AIHAs the immunologic signature ranges from overt antibody mediated autoimmunity against peripheral erythrocytes to a “central” autoimmune attack to bone marrow precursors. As a matter of fact, PNH clones, in the context of AA, are thought to be “surviving clones” spared after autoimmune attack against hematopoietic stem cells (8, 9); similarly, autoimmunity against erythroid precursors has been demonstrated in AIHA (3). Whether the emergence of PNH clones in AIHA is related to coexisting immune-mediated bone marrow failure and/or represents a favorable response to bone marrow stress related to acute hemolysis would require further investigation.

In conclusion, our data suggest testing PNH clones in AIHA since their prevalence is not negligible. Moreover, PNH positivity correlates with a prominent hemolytic pattern and may confer a higher thrombotic risk. Finally, cytokine patterns of PNH positive and negative AIHAs may give hints about the pathogenesis of highly hemolytic AIHA.

## Data Availability Statement

The datasets generated for this study are available on request to the corresponding author.

## Ethics Statement

The studies involving human participants were reviewed and approved by Ethical Committee of the Fondazione IRCCS Ca' Granda Ospedale Maggiore Policlinico. The patients/participants provided their written informed consent to participate in this study.

## Author Contributions

BF and WB designed the study, followed patients, collected and analyzed data, wrote the manuscript, and participated to the final revision. JG and AK followed patients, collected data, wrote the manuscript, and participated to the final revision. AZ performed the cytokine studies, wrote the manuscript, and participated to the final revision. LC performed the PNH clone testing. All authors participated to the design of the review, literature revision, manuscript writing, and final revision for important intellectual content.

## Conflict of Interest

The authors declare that the research was conducted in the absence of any commercial or financial relationships that could be construed as a potential conflict of interest.
